# Hybrid approach for long-standing persistent atrial fibrillation: immediate versus staged treatment

**DOI:** 10.1186/s13019-022-02019-x

**Published:** 2022-10-26

**Authors:** Giuseppe Nasso, Roberto Lorusso, Nicola Di Bari, Ignazio Condello, Felice Eugenio Agró, Flavio Fiore, Raffaele Bonifazi, Giuseppe Santarpino, Giuseppe Speziale

**Affiliations:** 1 Department of Cardiac Surgery, Anthea Hospital, GVM Care&Research, Via Camillo Rosalba, 35/38, 70124 Bari, Italy; 2 Cardio-Thoracic Surgery Department, Heart & Vascular Centre, Maastricht University Medical Centre (MUMC), Maastricht, Netherlands; 3 Cardiovascular Research Institute Maastricht (CARIM), Maastricht, Netherlands; 4 Department od Anaesthesiology, Campus Bio-Medico University, Rome, Italy; 5 Department of Experimental and Clinical Medicine, “Magna Graecia” University, Catanzaro, Italy; 6 Department of Cardiac Surgery, Paracelsus Medical University, Nuremberg, Germany; 7 Department of Cardiac Surgery, Città di Lecce Hospital, GVM Care&Research, Lecce, Italy

**Keywords:** Atrial fibrillation, Atrial fibrillation ablation, Bachmann’s bundle

## Abstract

**Background:**

The hybrid approach has become the most effective treatment option for restoring sinus rhythm and reducing the risk of atrial fibrillation (AF) recurrence. However, several issues remain to be clearly defined, including the appropriate timing of the staged procedure and the most effective strategy.

**Methods:**

Over a 12-year period of activity, we performed 609 AF ablation procedures via a right mini-thoracotomy. From this general population, 60 patients underwent a hybrid procedure with catheter ablation performed at least 4 weeks after the surgical procedure to confirm if effective complete electrical isolation of pulmonary veins was achieved. In 20 patients, the second stage procedure was performed during the same hospitalization due to patient’s electrical instability. The results obtained in immediate versus staged patients were compared.

**Results:**

All patients were discharged after the first stage procedure in sinus rhythm. The 20 immediate patients had a shorter hospital stay compared with the staged patients, in whom the two hospitalizations resulted in a longer hospital stay (immediate 5.5 ± 1.6 days versus staged 8.7 ± 1.4, P < 0.001). A significantly higher number of immediate patients had an associated ablation of the Bachmann’s bundle (n = 16 in the immediate group [80%] versus n = 14 in the staged group [45%]; P = 0.001). After a mean follow-up of 74 months, there was no significant difference in the risk of AF relapse between groups (immediate 1/20 [5%] versus staged 7/40 [17.5%]; P = 0.18).

**Conclusion:**

The hybrid approach for the treatment of AF was safe and effective in immediate restoring sinus rhythm and in its maintenance at follow-up. Our preliminary results show that both immediate and staged procedures show similar efficacy but this result is strongly influenced by the concomitant ablation of the Bachmann’s bundle, which appears to be the most important component of the treatment strategy in order to reduce the risk of recurrent AF.

## Introduction

The European Society of Cardiology (ESC) guidelines for the management of atrial fibrillation (AF) emphasize the importance of achieving complete electrical isolation of pulmonary veins (PV) during AF catheter ablation. However, this is often feasible only by combining surgical and endovascular strategies [1]. Although this hybrid approach has been widely used for the treatment of AF, many questions still remain open, particularly regarding the appropriate procedural timing. Most of the few available studies recommend a waiting time after the surgical procedure before endovascular treatment in order to favor scar formation and, hence, electrical isolation.

The appropriate timing, however, is not clearly addressed in current guidelines and the best treatment strategy for the individual patient should be selected after Heart Team discussion. In our experience in close cooperation with the Heart Team for the treatment of AF [2], patients were treated with either a hybrid approach with a staged strategy or a combined surgical and endovascular approach during the same hospitalization due to electrical instability after the surgical procedure.

The aim of this study is to report the outcome of the two different approaches to highlight a possible advantage of one of the two procedures. Our initial hypothesis was that patients with post-surgical electrical instability undergoing immediate endovascular treatment have a worse long-term outcome in terms of AF recurrence compared with patients undergoing a staged procedure.

## Methods

All study patients had AF lasting at least one year and none of these patients had previously undergone an isolated catheter ablation procedure. The outcome of our 2008–2020 case series, involving 609 consecutive patients undergoing surgical or two-staged hybrid ablation, has been described previously [3]. We also reported the outcome of patients treated with adjunctive Bachmann’s bundle (BB) ablation [2, 4]. The staged strategy agreed between cardiac surgeons and electrophysiologists was based on the hypothesis that a waiting time of at least 4 weeks after surgery before performing the catheter procedure would allow to confirm if effective complete electrical PV isolation was achieved, ultimately resulting in scar formation and atrial substrate change with elimination of AF triggers.

All patients underwent surgical AF ablation using a radiofrequency bipolar device (Estech COBRA Fusion™ 150 Surgical Ablation System). Among this population, 60 patients were evaluated and treated with a hybrid approach because the hybrid team (electrophysiologist and cardiac surgeon) tested this approach only in a period of the overall experience on the treatment of AF. Catheter ablation was performed under general anesthesia, esophageal temperature monitoring and using a tripolar catheter (Esotherm, Fiab). In addition to the closure of any gaps in the surgical ablation lines, all transcatheter procedures also involved ablation of the ligament of Marshall, roof and anterior mitral lines, coronary sinus and superior vena cava isolation, intercaval and cavotricuspid isthmus lines. Over time, as previously mentioned, the technology used has undergone changes in terms of the instrumental characteristics and the mode of energy supplied [2–4].

The 60 hybrid patients were surgically treated under general anesthesia, orotracheal intubation with selective mono-pulmonary ventilation to exclude the right lung, and were extubated on the operating table at the end of the procedure and admitted to the recovery/intensive care unit for 24 h post-procedure. Electrical instability consisting of high-rate atrial fibrillation or supraventricular tachyarrhythmia with impaired haemodynamics occurred early in 20 patients requiring drug therapy and leading the Heart Team to change the planned staged approach and to perform the second procedure during the same hospitalization. The parameters of the two groups (n = 20 “immediate” patients, n = 40 “staged” patients) were collected and compared. The two groups of patients do not differ in the use of antiarrhythmic therapy, size of the left atrium nor in BMI, and none of these patients had history of ischemic heart disease. The consumption of caffeine is given as positive based on the consumption of at least one cup of coffee / day and drinking based on the consumption of at least one glass of alcoholic beverage / day. All patients were referred to their cardiologists and followed up by phone interviews after the single admission in the “immediate” group or after the second admission in the “staged” group at 6, 9, 12 months and every 6 or 12 months thereafter, depending on rhythm stability.

The GVM Care & Research ethics committee approved the study and all patients provided written informed consent to the procedure and study enrollment.

### Statistical analysis

Categorical variables are given as counts and percentages. A p-value < 0.05 was considered statistically significant. Data analysis was performed using Excel 2016 (Microsoft, Redmond, WA, USA) and statistical analysis was performed using SPSS (IBM SPSS Statistics for Windows, Version 27.0; IBM Corp., Armonk, NY, USA).

## Results

The preoperative characteristics of the study population are reported in Table 1. The hybrid approach using a bipolar radiofrequency device was adopted in 60 patients adding a linear lesion targeting the BB in 30 patients (n = 16 in the immediate group [80%] versus n = 14 in the staged group [45%]; P = 0.001). The transcatheter procedure has always been preceded by a mapping for the evaluation of the surgical isolation of the pulmonary veins which revealed an absence of gap in all patients.

**Table 1:** Preprocedural characteristics of the study population

	Hybrid patients		P-value
	**Immediate** **(n = 20)**	**Staged** **(n = 40)**	
Age (years), mean ± SD	47 ± 11	39 ± 14	0.02
Ejection fraction (%),mean ± SD	54 ± 5	51 ± 6	0.052
NYHA class, mean ± SD	1.2 ± 0.4	1.1 ± 0.3	0.45
Smoking	7 (35%)	10 (25%)	0.41
Drinking	4 (20%)	6 (15%)	0.62
CPAP	2 (10%)	3 (7.5%)	0.74
Hypertension	10 (50%)	30 (75%)	0.052
Diabetes	2 (10%)	6 (15%)	0.59
Caffeine consumption	12 (60%)	5 (12.5%)	< 0.001

CPAP, continuous positive airway pressure; NYHA, New York Heart Association; SD, standard deviation

No intraoperative complications were recorded in either group at both stages. However, as per definition, the immediate group showed electrical instability as postoperative complication. In the staged group, no postoperative complications following both procedures were recorded. The 30-day mortality was 0% and no patient required pacemaker implantation. All patients who had undergone hybrid ablation either with or without BB ablation were discharged in sinus rhythm after completion of the first and second procedure.

The 20 immediate patients had a shorter hospital stay compared with the staged patients, in whom the two hospitalizations resulted in a longer hospital stay (immediate 5.5 ± 1.6 days versus staged 8.7 ± 1.4, P < 0.001).

After a mean follow-up of 74 months, there was no significant difference in the risk of AF relapse between groups (immediate 1/20 [5%] versus staged 7/40 [17.5%]; P = 0.18).

## Discussion

Monitoring of patients undergoing surgical or endovascular ablation of AF has evolved remarkably over the last years. Current guidelines emphasize the importance of achieving complete electrical PV isolation [1], a concept that cannot be taken for granted and should not be neglected given that PV reconnection rates are as high as 70% [1].

However, despite the large number of catheter ablation procedures, only few patients undergo multidisciplinary Heart Team discussion for proper decision making about hybrid AF ablation. Although various ablation strategies have been proposed and implemented into clinical practice [5–8], the success rate of catheter ablation in AF patients remains low for long-standing persistent AF, with wide variations in ablation techniques among operators. The results of our study suggest that the hybrid approach could be very effective in these patients, though being a more aggressive solution [9]. However, in our study, although all patients who had undergone two-staged hybrid ablation were discharged in sinus rhythm, we could not demonstrate the superiority of the hybrid approach over isolated surgical ablation. Indeed, in the staged group, all patients discharged after the first surgical step were in sinus rhythm. However, the different proportion of patients who had undergone adjunctive BB ablation in the two groups should also be considered, which represents an important limitation of our study.

The results of this study further support previous data from our group of a significant reduction of AF recurrence in hybrid patients in whom adjunctive BB ablation was performed [2]. However, we should reconsider our previous conclusions in that “BB ablation in the setting of a two-staged hybrid procedure is safe and highly effective” and “BB ablation does not increase the risk for periprocedural complications“[2]. Following data reanalysis in BB patients, 53% showed electrical instability postoperatively which was resistant to drug therapy (amiodaron and/or beta-blockers), requiring to change the treatment strategy (from staged to immediate). On the other hand, 80% of BB patients of the immediate group were able to maintain sinus rhythm at follow-up although the did not meet the criterion that led us to choose the staged procedure (scar formation). This could be due to the fact that the BB may be involved in a number of unstable reentrant circuits, and we hypothesized that an effective lesion in the BB would prevent induction and maintenance of AF, independent of scar formation.

Our study contributes to the direction to perform adjunctive BB ablation with the aim to improve the outcome and, in case of electrical instability, to perform immediately the second stage procedure without affecting the efficacy to reduce AF recurrence at follow-up.

Patients with hemodynamic instability after surgical ablation may have an arrhythmogenic substrate that explains this phenomenon but it is not information that we are able to provide to readers. In any case, even if this were the case, our early treatment could be proposed precisely for patients with this type of substrate given the safety and efficacy recorded.

As clear and for every article on AFib the possibility of recurrence would always be present in an asymptomatic form since patients are monitored in a discontinuous manner with telephone, electrocardiographic and clinical checks at intervals. However, it should be emphasized that the current guidelines consider the patient’s symptoms more important for the incidence of recurrence rather than the presence of an electrocardiographic alteration.

The main limitation of our study is that it included of a heterogeneous population of immediate/staged patients undergoing or not undergoing additional BB ablation. However, this is the largest case series to date of patients treated with both approaches. Further multicenter and randomized studies are warranted to confirm our results. In our opinion, although our study is not randomized, we comparable groups are needed to derive definite results and we believe that preoperative differences between groups do not affect the present results.

## Conclusion

Hybrid AF ablation is safe and effective using both the immediate and staged approach. The addition of BB ablation is associated with a risk of immediate electrical instability, but performing an immediate approach and discharging the patient in sinus rhythm results in a very low incidence of AF recurrence at follow-up.

## Data Availability

The datasets analyzed during the current study are available from the corresponding author on reasonable request.
